# gcMECM: graph clustering of mutual exclusivity of cancer mutations

**DOI:** 10.1186/s12859-021-04505-w

**Published:** 2021-12-14

**Authors:** Ying Hu, Chunhua Yan, Qingrong Chen, Daoud Meerzaman

**Affiliations:** Center for Biomedical Informatics and Information Technology, National Cancer Institute, Rockville, MD USA

**Keywords:** Cancer driver genes, Mutually exclusive mutations, Network analysis, R package

## Abstract

**Background:**

Next-generation sequencing platforms allow us to sequence millions of small fragments of DNA simultaneously, revolutionizing cancer research. Sequence analysis has revealed that cancer driver genes operate across multiple intricate pathways and networks with mutations often occurring in a mutually exclusive pattern. Currently, low-frequency mutations are understudied as cancer-relevant genes, especially in the context of networks.

**Results:**

Here we describe a tool, gcMECM, that enables us to visualize the functionality of mutually exclusive genes in the subnetworks derived from mutation associations, gene–gene interactions, and graph clustering. These subnetworks have revealed crucial biological components in the canonical pathway, especially those mutated at low frequency. Examining the subnetwork, and not just the impact of a single gene, significantly increases the statistical power of clinical analysis and enables us to build models to better predict how and why cancer develops.

**Conclusions:**

gcMECM uses a computationally efficient and scalable algorithm to identify subnetworks in a canonical pathway with mutually exclusive mutation patterns and distinct biological functions.

## Background

Next-generation sequencing technology has transformed the study of the cancer genome, enabling us to sequence whole-genome or whole-exome and measure somatic mutations in millions of cancer genomes. The Cancer Genome Atlas (TCGA), a publicly-funded genomics project, houses a collection of mutation profiles from thousands of patients and more than 30 different types of cancers [1]. Today’s comprehensive mutation landscape affirms the importance of identifying genes and their associated networks in the hunt for cancer driver genes. By detecting highly recurrent mutations, called “significantly mutated genes”, we can more reliably predict the development and trajectory of cancer. Finding these cancer-driver genes is difficult, and many simply escape identification using existing data sets and methods. For example, in breast cancer, only three genes (*TP53*, *PIK3CA* and *GATA3*) occur at > 10% incidence; for most tumor types, the current sample size is too small to reliably detect genes mutated at 5% or less above background mutation intensity [2]. Thus, we are not able to capture a complete representation of all genes and subsets of genes that drive the development and progression of cancers. Cancer genes tend to be altered in a finite number of pathways, typically related to differentiation, cell division, survival, and genomic maintenance [3]. Therefore, it is critical to identify pathway-level implications of genes, even those mutated at very low frequencies.

One approach to finding these drivers is to search for mutual exclusivity of altered genes since mutually exclusive pairs of genes often share the same pathways. For example, we know that a set of mutated genes rarely co-occurs in the same tumor and driver mutations are typically observed in exactly one gene in the pathway per patient [4]. The phenomenon could arise from functional redundancy or synthetic lethality in cancer pathways [5]. Classical examples of mutually exclusive driver mutations include EGFR and KRAS mutations in lung cancer [6] and TP53 and MDM2 mutations in glioblastoma [7]. Based on this rationale, MEMo (Mutual Exclusivity Modules in Cancer) draws on correlation analysis and statistical tests to identify network modules exhibiting patterns of mutually exclusive genetic alterations across multiple patients [8]. A more recent method Mutex uses a large, aggregated pathway model of human signaling processes to search groups of mutually exclusively altered genes, all of which share a common downstream event [9].

The drawback with the current methods is that they require extensive filtering of mutation data, which are limited to the most significantly mutated genes, focus on predefined network modules, and do not readily scale to reasonably sized datasets [10]. The mutual exclusivity signal can be biased toward identifying gene sets where the majority of the coverage comes from highly mutated genes [11, 12]. Although cancer genes have been shown to participate in multiple pathways, few existing methods identify polymorphic gene sets where a gene has different mutual exclusivity to other genes in different pathways at various mutation frequencies. To detect the mutually exclusive mutation pattern more broadly and capture the full range of mutations more comprehensively, we developed a novel graph-based unsupervised clustering approach to identify gene sets with mutually exclusive mutations. The Graph Clustering of Mutual Exclusivity of Cancer Mutations (gcMECM) is able to detect modules of different sizes with varying cutoffs and significance. The gene sets in a module can be mapped onto one or more canonical pathways to uncover functional subnetworks that can be associated with clinical features such as survival and tumor subtypes. The algorithm uses both high and low frequency mutations and is able to analyze a large set of genes.

## Implementation

### Mutation and pathway data

The mutation and clinical outcome datasets for TCGA-LUAD (Lung Adenocarcinoma) and TCGA-BRCA (Breast Invasive Carcinoma) in The Cancer Genome Atlas (TCGA) were downloaded from The NCI Genomic Data Commons (https://portal.gdc.cancer.gov, version 6). The missense, start_lost, stop_gained, and stop_lost mutations were included in the analysis due to the fact that single-nucleotide variants (SNVs) are the most reliable somatic mutation calls [13]. RAS pathway v2.0 is obtained from NCI Ras Initiative (ras-pathway-v2). The pathway structure and gene coordinates were created manually for visualization. KEGG pathway images and gene relationships were from KEGG database (https://www.genome.jp/kegg).

### Detection of modules with mutually exclusive mutations

**Fig. 1:**
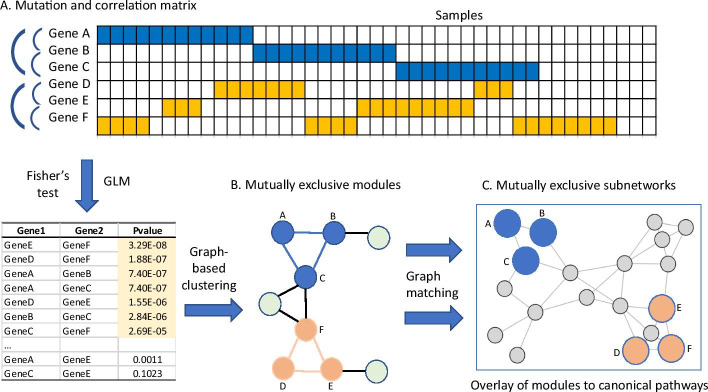
Schematic diagram of gcMECM. **A** The mutation matrix, displaying a landscape of mutation status across genes at vertical axis and samples at horizontal axis, is used to calculate gene–gene association of mutations with Fisher’s exact test and generalized linear models (GLM). Arches are used to highlight subnetworks. **B** Identification of modules with graph-based clustering of negatively correlated genes. **C** Overlay of mutually exclusive subnetworks in the context of canonical pathways using the graph-matching algorithm

gcMECM was implemented in R (http://www.R-project.org) and available on GitHub at https://github.com/CBIIT-CGBB/gcMECM. Its workflow consists of three steps (Fig. 1). First, it generates the association matrix or gene–gene adjacency distance matrix using Fisher's exact test and generalized linear models (GLM), which are performed for every pair of genes in the mutation matrix across all tumor samples. As shown in the schematic diagram (Fig. 1A), genes A-B-C and genes D-E-F have strong negative associations while the association between genes A and D is weak.

Secondly, gcMECM constructs network graphs from a set of negatively correlated genes selected from those with both negative correlations in the GLM and Fisher’s exact test p value < 0.001 (Fig. 1B). This p-value is used as a distance between genes and can be set with a less stringent value to build an inclusive graph. As long as the p-value in this step is relaxed enough to retain those potential mutually exclusive mutation gene pairs, slightly different p-value selection will not affect the downstream analysis. The graph is subsequently clustered into modules by Louvain algorithm in the R package igraph (https://igraph.org). The Louvain algorithm can quickly detect modules in a large graph based on the modularity measure and a heuristic approach [14] and is one of the most popular algorithms in the biological network analysis [15]. By applying the stringent cutoff of edge values less than 1.00E−08 and 1.00E−12 in TCGA-BRCA and TCGA-LUAD respectively, genes in each module are closely related to each other with negative relationships due to the mutually exclusive mutations. The edge cutoff p-values were chosen to generate 5–10 moderate size modules, which are computationally efficient while maintain the biological function integrity. The number of genes in a moderate size module is expected to be around hundreds, similar to that in RAS and KEGG pathways. Modules with less than three genes were removed from further analysis.

Finally, each module is compared to canonical pathways from NCI Ras or KEGG database to identify subnetworks via a graph matching algorithm in the R package igraph [16]: 1) determine subnetworks in a module consisting of the common genes between a module and a canonical pathway; 2) genes in a subnetwork must have a minimum of three genes with direct connections in the canonical pathway. This is to ensure the subnetwork from a module is localized and does not spread across the pathway. A combined p-value for a subnetwork is calculated from gene-pair Fisher’s exact test p-values using combine.test function in the R package survcomp. All subnetworks in the context of pathways are visualized with the R package igraph and further examined for the enrichment with g:profiler (https://biit.cs.ut.ee/gprofiler/gost) and OmicPath (https://github.com/CBIIT-CGBB/OmicPath) against Gene Ontology and KEGG. The survival analysis is carried out with the R survival package.

## Results

To demonstrate the utility and performance of gcMECM, we analyzed the missense, start_lost, stop_gained, and stop_lost mutations from TCGA breast invasive carcinoma (TCGA-BRCA) and lung adenocarcinoma (TCGA-LUAD) to identify modules with mutually exclusive mutation patterns [17, 18]. Using TCGA-BRCA data, we identified 9451 genes mutated in 985 samples. Of these, 3784 genes have negative correlations (Fisher’s exact test p value < 0.001). A total of 6 modules were detected with the minimal module size of 155 genes and the maximal module size of 1106 genes. Similarly, using TCGA-LUAD data, we found 12,683 genes mutated in 565 samples, with 4440 genes having negative correlation (Fisher’s exact test p value < 0.001). A total of 7 modules were identified with the minimal module size of 85 genes and the maximal module size of 1114 genes.

We next mapped modules from TCGA-LUAD and TCGA-BRCA onto the Ras pathway to identify subnetworks, which reduced the complexity and could be used for the detection of biologically relevant patterns. This pathway is critical in carcinogenesis and includes genes involved in oncogenic signaling, cell cycle, DNA replication, and DNA repair. Those genes are frequently altered in different cancers, including AKT1, EGFR, KRAS, and STK11 in lung cancer and AKT1, BRCA2, ERBB2, and PIK3CA in Breast cancer [19].

**Fig. 2:**
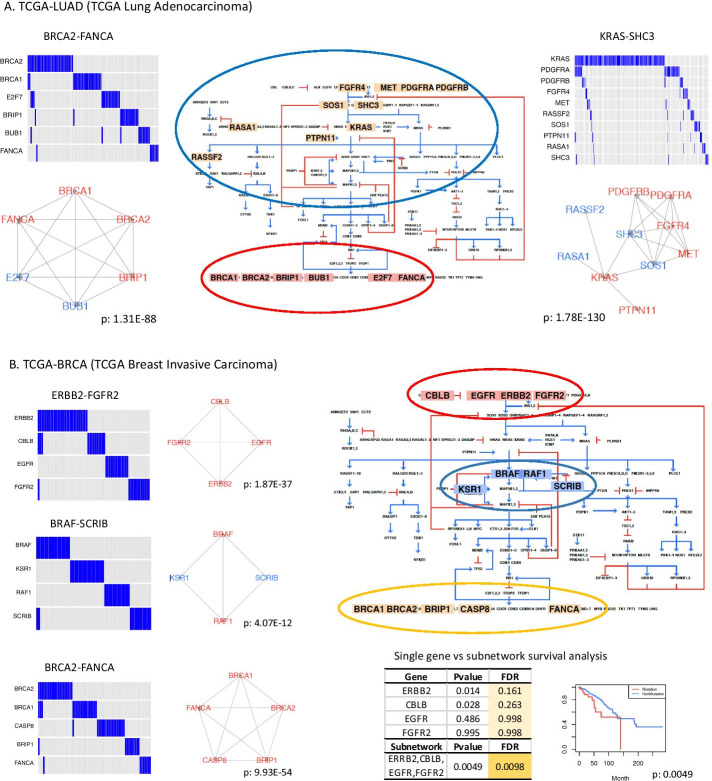
Biological implication of subnetworks in the context of pathway. **A** Subnetworks from TCGA-LUAD (TCGA Lung Adenocarcinoma) are mapped to the RAS pathway. Genes colored in red are present in the COSMIC Cancer Gene Census. Blue oval: genes in KRAS-SHC3; Red oval: genes in BRCA2-FANCA. **B** Subnetworks from TCGA-BRCA (TCGA Breast Invasive Carcinoma) are mapped to the RAS pathway and associated with survival. Red oval: genes in ERBB2-FGFR2; Blue oval: genes in BRAF-SCRIB; Yellow oval: genes in BRCA2-FANCA. P-values are combined p-values from gene-pair fisher’s tests. The KM-plot of survival analysis is obtained from the comparison of mutated vs. non-mutated samples for four genes in the subnetwork ERBB2-FGFR2. Coxph p-values adjusted by sex and age for both single gene and a group by subnetwork genes are shown in the table

Two subnetworks in TCGA-LUAD, KRAS-SHC3 and BRCA2-FANCA, have been shown to have mutually exclusive mutations and more than half of those genes are present in COSMIC cancer census genes [20] (Fig. 2A). Most genes have a low mutation rate; only KRAS and PDGFRA have a mutation frequency greater than 5%. The KRAS-SHC3 subnetwork captures the upstream signaling component in the RAS pathway, which involves the ERBB signaling pathway, VEGF-PDGFR signaling pathway, and MAPK signaling pathway, as demonstrated using the Gene Ontology and KEGG analyses with g:Profiler [21]. The BRCA2-FANCA subnetwork is related to meiotic cell cycle process, cell signal transduction by p53 class mediator, DNA replication, and homologous recombination. These two subnetworks with distinct biological functions suggest that the mutual exclusivity in genes with related functionality could be used to identify cancer-relevant genes, especially when the subnetworks also include well-established cancer genes.

Three subnetworks, ERBB2-FGFR2, BRAF-SCRIB, and BRCA2-FANCA, are identified in TCGA-BRCA after mapping to RAS pathway (Fig. 2B). The ERBB2-FGFR2 subnetwork is related to ERBB signaling pathway and BRCA2-FANCA subnetwork is linked to DNA repair and meiotic cell cycle process, which are similar to KRAS-SHC3 and BRCA2-FANCA subnetworks in TCGA-LUAD respectively. The BRAF-SCRIB subnetwork is found to regulate MAP kinase activity and ErbB signaling pathways that are linked to many cancers such as melanoma, lung, ovarian, breast, and prostate [22]. The mutation rate of genes in these three subnetworks is all less than 5%. Each subnetwork consists of genes with similar functions, which can be used to group samples into mutated and non-mutated categories for survival analysis. As seen in ERBB2-FGFR2 subnetwork, the survival difference is not statistically significant between two groups at the individual gene level. However, if samples are divided into two groups based on the mutation status of all genes in ERBB2-FGFR2 subnetwork, the mutation group exhibits a significantly lower survival (FDR < 0.05). These results demonstrate that integrating subnetworks of mutually exclusive mutations with pathways and clinical features can aid in interpreting the subnetwork’s resulting biological functions.

## Conclusions

gcMECM expands the current mutual exclusivity software capability into studying genes with low frequency mutation and data with large sample sizes through the integration of graph modules, canonical pathways, and clinical information. It uses a computationally efficient Louvain algorithm, which is readily scalable to handle a large number of genes, samples, and networks. The edge cutoff p values as parameters are subject to tuning since mutation types and mutation rates have an impact on the module and subnetwork structure. For example, the current use case consists of functional SNVs. If frameshift mutations were included, subnetwork structures would be partially altered due to the change of mutation frequency. As shown here, the mapped subnetworks are suitable for gene set enrichment analysis to identify functionally coherent gene groups which are particularly important in exploring functional redundancy or synthetic lethality in cancer pathways [5]. Depending on the canonical pathway, a single gene may be instrumental in multiple subnetworks in cancer, with very distinct biological functions. Thus, examining the subnetwork, and not just the impact of a single gene, should significantly increase the statistical power of clinical analysis.

## Availability and requirements

Project name: gcMECM; Project home page: https://github.com/CBIIT-CGBB/gcMECM; Operating system(s): Platform independent; Programming language: R; Other requirements: R (> = 3.5), igraph, Rtsne; License: GNU GPL v2.0; Any restrictions to use by non-academics: No.

## Data Availability

gcMECM R package is available at https://github.com/CBIIT-CGBB/gcMECM; Ras pathway was downloaded from https://www.cancer.gov/research/key-initiatives/ras/ras-central/blog/2015/ras-pathway-v2; TCGA breast cancer and lung cancer mutation data were obtained from https://portal.gdc.cancer.gov.

## Abbreviations

gcMECM: Graph Clustering of Mutual Exclusivity of Cancer Mutations
TCGA: The Cancer Genome Atlas
BRCA: Breast invasive carcinoma
LUAD: Lung Adenocarcinoma
KEGG: Kyoto Encyclopedia of Genes and Genomes
GO: Gene Ontology
COSMIC: The Catalogue of Somatic Mutations in Cancer
FDR: False discovery rate
GLM: Generalized linear model
